# CX3CR1-T280M polymorphism and end-stage renal disease development in chronic kidney disease

**DOI:** 10.1038/s41598-026-40013-8

**Published:** 2026-04-03

**Authors:** Matteo Gatti, Ivano Baragetti, Andrea Baragetti, Laura Buzzi, Andrea Lupi, Angelo Fassio, Elena Olmastroni, Alberico L. Catapano, Paolo Fabbrini

**Affiliations:** 1 Department of Nephrology and Dialysis Ospedale Bassini, ASST Nord Milano-Cinisello Balsamo, Via M. Gorki 50, 20092, Cinisello Balsamo, 20092 Italy; 2 Department of Pharmacological and Biomolecular Sciences Rodolfo Paoletti, University of Milan, Milan, Italy; 3 IRCCS MultiMedica, Sesto San Giovanni (MI), Sesto San Giovanni, Italy; 4 Epidemiology and Preventive Pharmacology Service (SEFAP), Department of Pharmacological and Biomolecular Sciences, University of Milan, Milan, Italy; 5 Rheumatology Unit, University of Verona, Verona, 37134 Italy

**Keywords:** Chronic inflammation, Chronic kidney disease, CX3CR1-CX3CL1 (fractalkine), Dialysis, Monocytes, Single-nucleotide polymorphism, Molecular medicine, Risk factors, Genetics research, Outcomes research, Renal replacement therapy, Atherosclerosis, Nephrosclerosis, Diseases, Kidney diseases, Chronic kidney disease, End-stage renal disease, Nephrosclerosis, Nephrology, Kidney diseases, Chronic kidney disease, End-stage renal disease, Clinical genetics, Immunopathogenesis, Pathogenesis, Chronic inflammation, Diabetes complications

## Abstract

Chronic kidney disease (CKD) is a major global health concern driven by hypertension and diabetes, with genetic factors playing a key role in its pathogenesis. CX3CR1, the sole known receptor for the chemokine fractalkine, and its polymorphisms T280M and V249I have been implicated in the progression of several chronic diseases. This prospective observational study investigates the role of the CX3CR1 T280M polymorphism in CKD progression to end-stage disease. We included 121 CKD patients with varying renal insufficiency severity. Patients were stratified by CX3CR1-T280M genotype: Group 1 wild-type (T/T, 71.9%) and Group 2 carriers of the mutated allele (T/M or M/M, 28.1%). The primary outcome was kidney replacement therapy (KRT) initiation over 18 years. Cox proportional hazards univariate analysis was used for survival assessment. The M allele for CX3CR1 is associated with higher baseline serum creatinine levels, consistent with previous cross-sectional studies. During follow-up, KRT was initiated in 26 patients (17.2% in Group 1, 32.3% in Group 2). Survival analysis showed a significant association between the CX3CR1-T280M polymorphism and CKD progression (HR 2.28, 95% CI: 1.04–4.98, p 0.039). This study confirms the role of the CX3CR1-T280M polymorphism in determining prognosis in CKD patients, particularly in predicting the risk of a critical outcome such as initiation of KRT.

## Introduction

Chronic kidney disease (CKD) is the third fastest growing cause of death globally, due to an aging population and the rising incidence of hypertension and type 2 diabetes mellitus^1^. These two conditions are the primary causes of CKD; however, the progressive decline in renal function involves a complex and multifactorial pathogenesis^2^. Among the various factors involved, genetics certainly plays a significant role. Research in this field has already provided substantial benefits in terms of diagnosis and therapy for various forms of CKD in selected patients^3^. Low-grade systemic inflammation also contributes significantly to CKD development and progression, though the precise timeline of this process remains incompletely defined^4^. Inflammatory cytokines and chemokines—produced primarily by monocytes and endothelial cells—are central mediators in this context^5,6^.

Among inflammatory mediators, the receptor for advanced glycation end products (RAGE) has been suggested to play a role in CKD pathogenesis^7,8^. Activation of RAGE by AGEs promotes oxidative stress, cytokine release, and endothelial dysfunction—hallmarks of progressive renal and cardiovascular damage. A functional polymorphism in the promoter region of the RAGE gene (− 374 T/A) has been associated with increased transcription and possibly with a reduced risk of microvascular complications, though data across studies remain inconsistent^9^. This genetic variability may influence the balance between membrane-bound and soluble RAGE, potentially modifying individual susceptibility to renal injury.

C-X3-C motif chemokine ligand 1 (CX3CL1) is another chemokine of particular interest. It is the only member of the CX3C subfamily, firstly identified by two separate research groups in 1997 and named fractalkine and neurotactin by the authors independently^10,11^. It was identified in most human tissues with the exception of peripheral blood cells, and since its discovery a pro-inflammatory role was suggested due its upregulation in the brains of mice with experimental encephalitis.

CX3CL1 differs from all other chemokine for a unique cysteine pattern (Cys-X-X-X-Cys) and its characteristic structure comprised of CX3C domain and a mucin stalk^10^. In the same year of its discovery Imai et al. demonstrated that CX3CL1 binds with high affinity the orphan V28 receptor, a G protein coupled receptor (GPCR) that was subsequently renamed CX3CR1^12^. The active CX3C domain of CX3CL1 may be released as a soluble chemokine or may be expressed at the cell surface via the transmembrane mucin stalk to functions as adhesion molecule via binding CX3CR1^10^. G protein signaling is necessary for CX3CR1 to induce migration, but not to support adhesion; interesting, CX3CR1 remains the only receptor through which CX3CL1 interact^12^.

More recent studies have demonstrated that CX3CR1 is widely expressed in human tissues, especially in mononuclear myeloid compartment but also by NK cell subset and T-cell populations^12–15^. The expression is often highly cell type-specific: in the brain is restricted to microglia, in the gut is limited to lamina propria macrophages, in the blood is mainly restricted to monocytes^16,17^.

In the kidney CX3CR1 is expressed in glomeruli, tubular epithelial cells, and peritubular capillaries during condition with significant mononuclear leukocyte infiltration, like acute crescentic glomerulonephritis or acute renal allograft rejection. This expression is absent in normal kidneys and non-inflammatory conditions such as minimal change disease^18^. Additionally, the majority of cells infiltrating the glomeruli and tubulointerstitium in various kidney diseases express CX3CR1, with this expression not limited to specific cell subsets or localized environments within the kidney during inflammatory conditions^19^.

Two common single nucleotide polymorphisms (SNPs) have been identified in the CX3CR1 open reading frame. These nonsynonymous substitutions result in conservative amino acid changes in the CX3CR1 protein: replacing of valine by an isoleucine at position 249 (V249I), and replacing of methionine by a threonine at position 280. Both SNPs exhibit strong linkage disequilibrium, forming the prevalent I249M280 haplotype^20^. The CX3CR1-M280 haplotype shows reduced binding affinity to CX3CL1 in circulating monocytes (PBMCs), as both in vivo and in vitro. These studies also indicate that this haplotype correlates with faster disease progression in AIDS patients^21 ^and reduced adhesion and chemotaxis activity, which is associated with a lower risk of cardiovascular disease in the Framingham Offspring Study cohort^22^. Taken together, these findings suggest that CX3CR1 may influence the pathogenesis of various diseases through its role in fractalkine-mediated inflammatory events. The CX3CL1-CX3CR1 axis and its polymorphisms appear to play significant roles in inflammatory processes underlying multiple diseases, including CKD. Given the complexity of pathogenic mechanisms leading to end-stage renal disease (ESRD), our study aim to prospectively explore the potential role of CX3CR1 and RAGE gene polymorphisms in the progression of renal insufficiency.

## Results

**Fig. 1:**
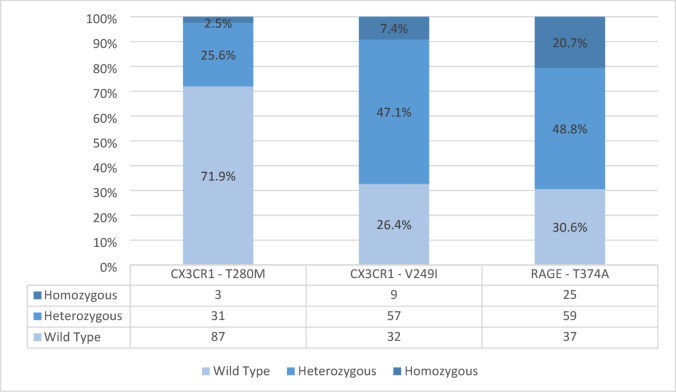
*Prevalence of CX3CR1 and RAGE genes polymorphisms*. * Due to the small number of homozygotes for the T280M polymorphisms of the CX3CR1 gene*,* these were combined with heterozygotes for statistical analyses*.

Overall, 121 patients were enrolled; the median (25th, 75th percentile) follow-up time was 125 (89, 193) months. The distribution of the T280M and V249I polymorphisms for the CX3CR1 gene and the T374A polymorphism for the RAGE gene are presented in Fig. 1. Note that the analysis of the V249I polymorphism is available for only 98 of the 121 recruited patients (81%).

The baseline anthropometric and biohumoral characteristics of patients in Group 1 and Group 2 are presented in Table 1.

**Table 1:** *Baseline characteristic of the population according to T280M genotype*. *The anthropometric parameters*,* the prevalence of past cardiovascular events (myocardial infarction*,* acute coronary syndrome*,* stroke*,* transient ischemic attacks*,* by-passes at the inferior limbs*,* angioplasty*,* aorto-coronary by-passes) and biohumoral parameters are compared between patients carrying the 280 T/T (Group 1) and T/M + M/M (Group 2) genotypes of CX3CR1.*

Parameter	Group 1 [TT] mean ± SD [CI]	Group 2 [TM/MM] mean ± SD [CI]	Chi square/T-test/Mann-Whitney U test	Significance (*p*)
SEX (n° M/F)	59/28	26/8	0.876	0.349
Smoke (n° - %)	13 (14.9%)	4 (11.7%)	0.204	0.651
Diabetes type 2 (n° - %)	60 (68.9%)	26 (76.4%)	0.670	0.413
Past cardiovascular event (n° - %)	21 (24.1%)	11 (32.3%)	0.848	0.357
Age (years)	65.26 ± 6.84 [61.31–69.21]	67.33 ± 10.67 [54.08–80.58]	−1.051	0.295
BMI (Kg/m^2^)	29.64 ± 5.52 [26.45–32.83]	35.19 ± 8.14 [25.08–45.3]	−0.759	0.454
Mean arterial pressure (mmHg)	96.71 ± 12.27 [89.63–103.80]	103.40 ± 7.40 [94.21–112.59.21.59]	0.056	0.955
Creatinine (mg/dl)	1.44 ± 0.63 [1.31–1.58]	1.84 ± 0.99 [1.49–2.20]	2.107	**0.035**
Urea (mg/dl)	66.04 ± 42.91 [41.27–90.82]	54.8 ± 31.84 [15.26–94.34]	−0.559	0.576
Calculated GFR CKD-EPI 2021 (ml/min)	59.83 ± 28.77 [53.59–66.08]	47.42 ± 23.98 [38.92–55.93]	2.273	**0.025**
Albuminuria (mg urinary albumin/mmol urinary creatinine)	29.50 ± 30.66 [11.80–47.20]	24.40 ± 20.24 [0–49.54.54]	−0.596	0.553
Hemoglobin (g/L)	130.0 ± 12.4 [122.8–137.2.8.2]	140.0 ± 15.8 [120.4–159.6.4.6]	0.151	0.880
Platelet (10^9/L)	214.57 ± 46.79 [187.55–241.59.55.59]	245.00 ± 67.23 [161.52–328.48.52.48]	−0.475	0.636
Sodium (mmol/L)	134.2 ± 4.9 [133.0–135.5.0.5]	133.5 ± 4.8 [131.4–135.6.4.6]	−0.618	0.537
Potassium (mmol/L)	4.43 ± 0.64 [4.06–4.80]	4.40 ± 0.54 [3.72–5.08]	0.390	0.698
Ca x P product (mg/dl)	33.23 ± 6.78 [29.31–37.15]	30.68 ± 3.27 [26.61–34.74]	0.790	0.431
Parathyroid hormone (pg/ml)	86.69 ± 12.20 [62.0–111.3.0.3]	88.0 ± 19.70 [45.7–130.2.7.2]	−0.56	0.956
Uric acid (mg/dl)	6.42 ± 1.44 [5.58–7.25]	5.04 ± 1.44 [3.24–6.83]	0.688	0.494
HbA1c (%)	7.12 ± 0.84 [6.64–7.61]	7.54 ± 0.78 [6.56–8.51]	−0.593	0.555
Total cholesterol (mg/dl)	182.21 ± 46.18 [155.55–208.88.55.88]	201.60 ± 42.98 [148.23–254.97.23.97]	0.212	0.833
LDL cholesterol (mg/dl)	102.34 ± 40.0 [79.24–125.44.24.44]	115.76 ± 37.68 [68.96–162.55.96.55]	−0.531	0.596
HDL cholesterol (mg/dl)	46.21 ± 12.23 [39.15–53.28]	50.20 ± 7.85 [40.45–59.95]	1.614	0.109
Triglycerides (mg/dl)	168.64 ± 91.83 [115.62–221.67.62.67]	178.0 ± 57.29 [106.86–249.14.86.14]	0.733	0.465
Interleukin 6 (pg/ml)	27.03 ± 65.67 [10.21–43.85]	22.81 ± 30.84 [11.30–34.33.30.33]	1.710	0.087
Interleukin 8 (mg/dl)	60.99 ± 85.77 [37.80–84.17.80.17]	28.50 ± 21.29 [19.29–37.71]	−1.896	0.058
Serum RAGE (pg/ml)	1749.3 ± 850.5 [1258.2–2240.4.2.4]	1221.8 ± 413.9 [707.8–1735.7.8.7]	0.156	0.876

Within the cohort of 121 enrolled patients, stratification according to the T280M polymorphism of the CX3CR1 gene identifies 87 patients with the T/T genotype (Group 1; 71.9%) and 34 patients with the T/M or M/M genotypes (Group 2; 28.1% of the total). Specifically, Group 2 included 31 heterozygous T/M patients (25.6%) and 3 homozygous M/M patients (2.5%). No significant differences among the genotypes were observed according to age, sex, mean arterial pressure, smoking habits, presence of diabetes or previous cardiovascular events. Electrolytes assessments, calcium-phosphorus balance, haemoglobin, platelet count, uric acid, glycosylated hemoglobin, total cholesterol, HDL and LDL cholesterol, triglycerides, interleukin 6, serum RAGE were similar among the two groups, too. Interleukin 6 and interleukin 8 plasma levels of Group 1 patients were higher (27.03 versus 22.81 and 60.99 versus 28.50, respectively), but differences were only of borderline statistical significance (p 0.087 and p 0.058, respectively).

The serum creatinine levels at the time of enrollment were 1.44 mg/dl (95% CI 1.31 to 1.58) for Group 1 and 1.84 mg/dl (95% CI 1.49 to 2.20) for Group 2. The difference between the two groups was statistically significant (*p* = 0.035). Similarly, the estimated glomerular filtration rate (eGFR) calculated using the CKD-EPI 2021 formula was significantly different between the two groups: 59.83 ml/min (95% CI 53.59 to 66.08) for Group 1 versus 47.42 ml/min (95% CI 38.92 to 55.93) for Group 2 (*p* = 0.025).

Table 2 presents the pharmacological history at the time of enrollment. All patients were treated according to clinical judgment and in adherence to current guidelines^23,24^. No differences were observed between the two groups in the use of antiplatelet, antihypertensive, oral antidiabetic drugs or insulin. However, patients in Group 1 had a higher usage of lipid-lowering drugs (36.8% versus 14.7%, *p-value* 0.018) and uric acid-lowering drugs (24.1% versus 5.9%, *p-value* 0.021).

**Table 2:** *Comparison of therapy at enrollment between patients carrying the 280 T/T (Group 1) and T/M + M/M (Group 2) genotypes of CX3CR1*.

Medication	Group 1 [TT] number (%)	GROUP 2 [TM/MM] number (%)	Pearson chi-square	Significance (*p*)
ASA/Ticlopidine	41 (47.1%)	10 (29.4%)	3.146	0.076
Allopurinol	21 (24.1%)	2 (5.9%)	5.292	*0.021*
Statins or fibrates	32 (36.8%)	5 (14.7%)	5.612	*0.018*
Beta-blockers	18 (20.7%)	6 (17.6%)	0.142	0.706
Clonidine	2 (2.3%)	2 (5.9%)	0.982	0.322
Alfa-antagonist	9 (10.3%)	3 (8.8%)	0.063	0.801
Calcium channel blocker	19 (21.8%)	9 (26.5%)	0.295	0.587
Diuretics	33 (37.9%)	7 (20.6%)	3.323	0.068
ARBs	24 (27.6%)	9 (26.5%)	0.015	0.901
ACE inhibitors	38 (43.7%)	14 (41.2%)	0.062	0.803
Oral antidiabetics	36 (41.4%)	14 (41.2%)	0.000	0.984
Insulin	15 (17.2%)	4 (11.8%)	0.554	0.457

Over the 18-year observation period, a total of 26 patients initiated KRT. Of these, 15 events occurred within Group 1 (involving 15 out of 87 patients, 17.2%) and 11 events were recorded in Group 2 (involving 11 out of 34 patients, 32.3%).

**Fig. 2:**
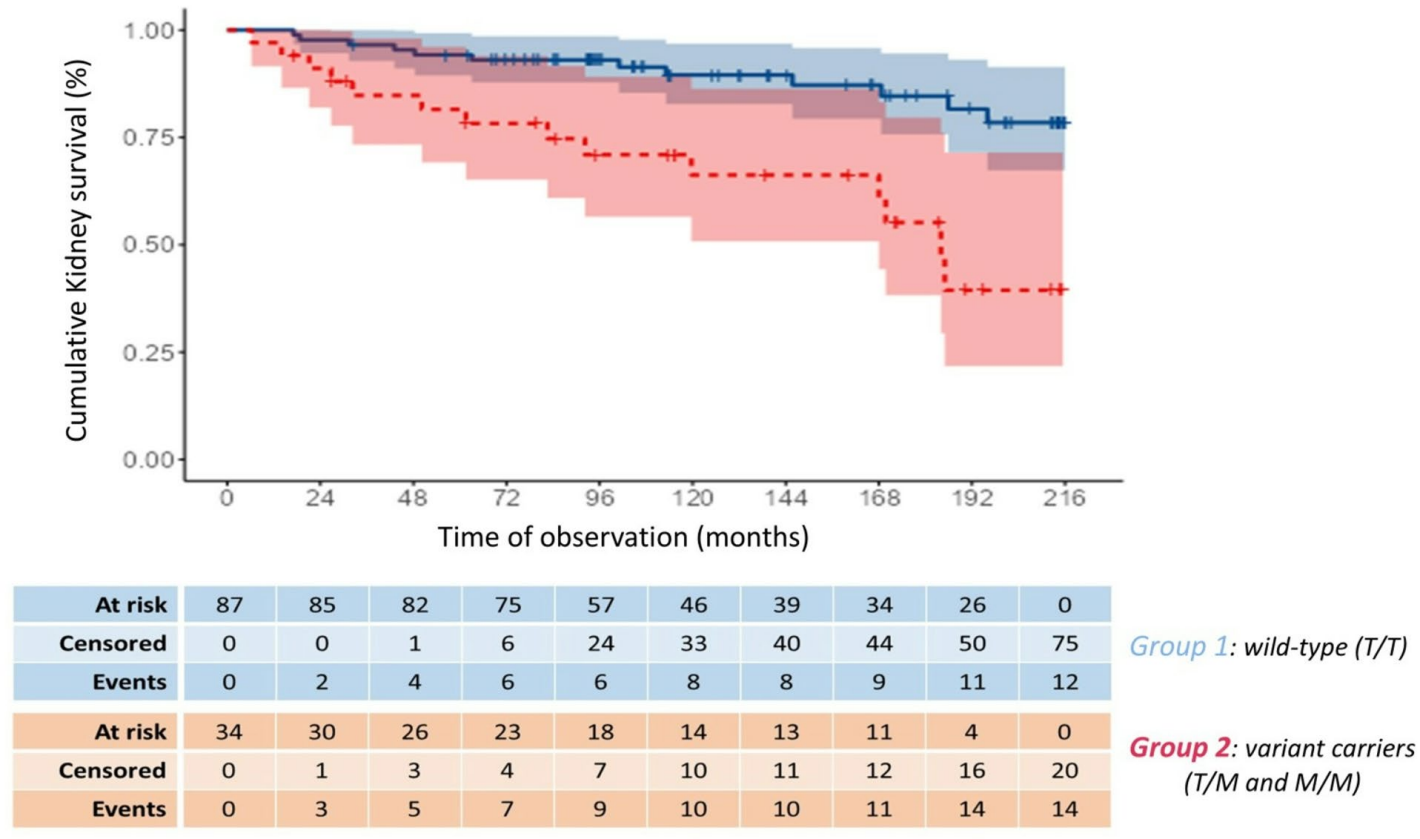
Kaplan-Meier analysis of renal survival in patients carrying − 280 T/T genotype and the M allele. Subjects carrying the M allele (Group 2) present a greater incidence of KRT compared with wild type subjects (Group 1). Overall, 26 KRT events were reported: 15 in T/T patients, 11 in T/M or M/M patients. The survival table is displayed below the curves.

Survival analysis indicates that the T280M polymorphism of the CX3CR1 gene correlates with progression of CKD to end-stage renal disease (Fig. 2). Patients in Group 2 carrying the M allele in heterozygous (T/M) or homozygous (M/M) form have a higher risk of requiring KRT compared to Group 1 patients with the T/T genotype (HR 2.28, 95% CI 1.04 to 4.98; *p* 0.039).

Survival analysis did not demonstrate an association between carriers of the V249I-CX3CR1 mutant allele and the initiation of KRT (*p* 0.621). No correlation was found even in analyses conducted on V249I-CX3CR1 wild-type versus heterozygous and homozygous combined groups (*p* 0.741). Similarly, T374A-RAGE was not associated with the initiation of KRT, both in survival analysis for mutant allele carriers (*p* 0.395) and in the wild-type versus heterozygous and homozygous combined analysis (*p* 0.066).

The multivariate analysis, which included T280M-CX3CR1, V249I-CX3CR1, and T374A-RAGE as covariates, confirmed the previously described results: T280M-CX3CR1 plays a statistically significant role, while the other two polymorphisms showed no correlation with the outcome.

A multivariate analysis was subsequently performed including T280M-CX3CR1, V249I-CX3CR1, and T374A-RAGE polymorphisms, as well as age, sex, mean arterial pressure, albuminuria levels, total cholesterol and eGFR at the time of enrollment as covariates. Among these, albuminuria levels (HR 1.03, 95% CI 1.01 to 1.06; p 0.007), total cholesterol (HR 1.02, 95% CI 1.01 to 1.03; p 0.005), and the T280M-CX3CR1 polymorphism (HR 3.86, 95% CI 1.08 to 13.75; p 0.037) showed a statistically significant association with the primary outcome.

**Fig. 3:**
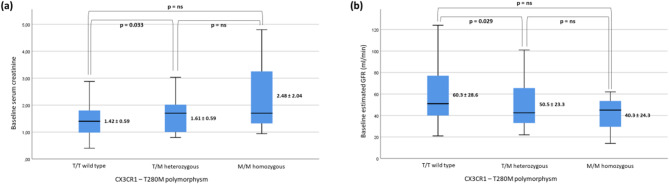
In depth analysis of baseline renal function based on T280M genotype. Serum creatinine **(a)** and eGFR calculated with CKD-EPI 2021 formula **(b)** are compared between patients carrying the 280 T/T wild-type, heterozygous T/M and homozygous M/M.

The cross-sectional analysis in Fig. 3 explore the association between age and eGFR at baseline. Patients with the T/T genotype exhibit preserved baseline renal function (creatinine 1.42 mg/dl and eGFR 60.3 ml/min) compared to patients with the heterozygous T/M genotype (creatinine 1.61 mg/dl and eGFR 50.5 ml/min), and even more so compared to patients with the homozygous M/M genotype (creatinine 2.48 mg/dl and eGFR 40.3 ml/min).

**Fig. 4:**
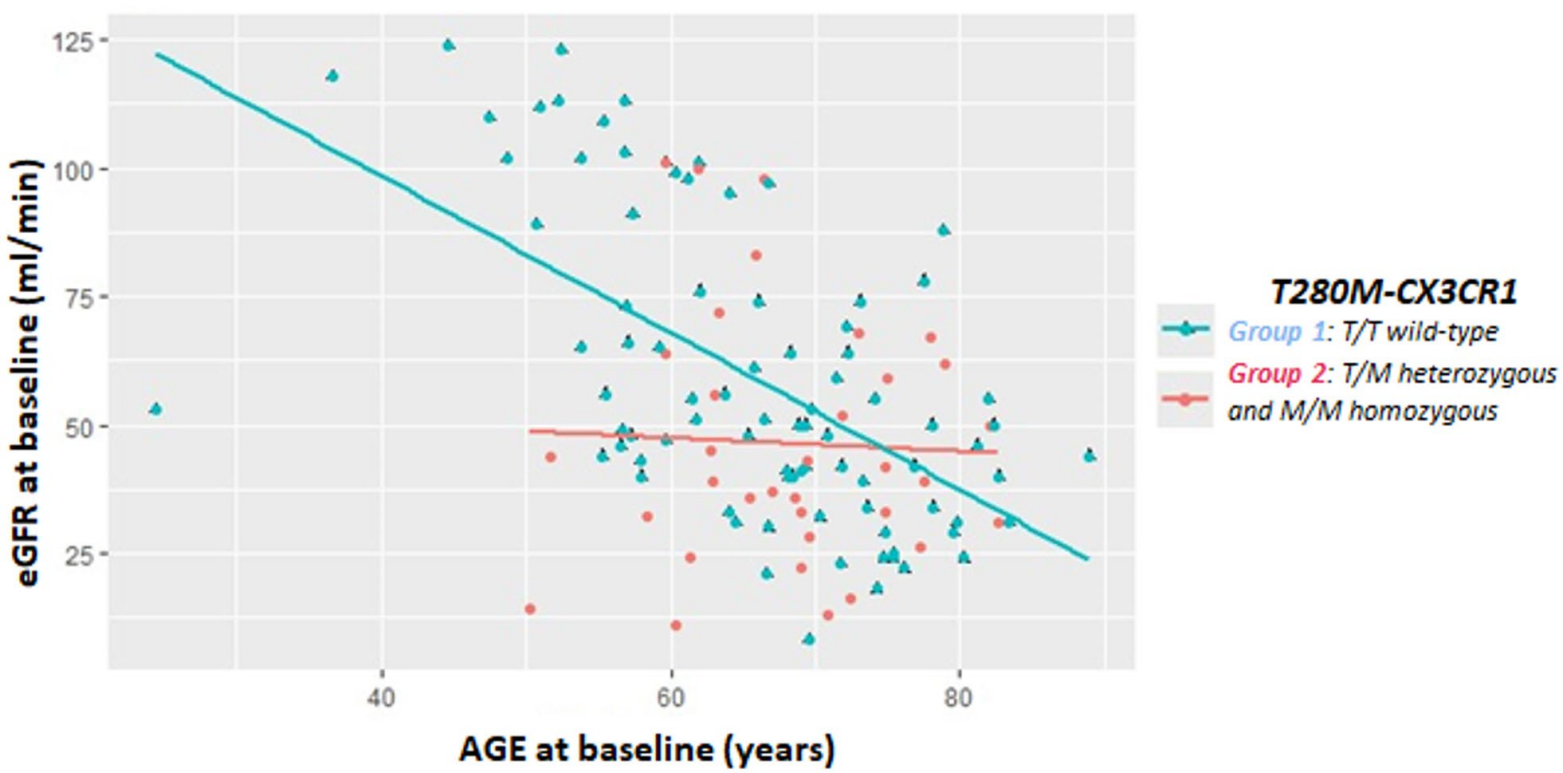
Scatter plot illustrating the relationship between age at enrollment and baseline eGFR according to T280M genotype. The interaction term in the regression analysis was statistically significant (*p* = 0.017), indicating that the slope of eGFR decline with age differs between the two groups.

The linear regression model for eGFR in Fig. 4 show an estimated annual reduction of 1.52 ml/min/m^2^ (CI −1.99; −1.06, *p* < 0.001) only for Group 1 patients; no relationship between age and eGFR at enrollment was observed for Group 2 patients (CI −1.23; 0.97, *p* = 0.81).

## Discussion

Our results show that CKD patients carrying the M allele for the T280M polymorphism of the CX3CR1 gene have a poorer renal prognosis compared to those with the T/T (wild-type) genotype. This relationship remains consistent following multivariate adjustment for major clinical determinants of CKD progression, namely total cholesterol, urinary albumin levels and mean arterial pressure.

To our knowledge, this is the first prospective study to demonstrate this correlation. The significance of this finding is underscored by the fact that the initiation of renal replacement therapy is a hard outcome, and we observed this association in an unselected group of patients with a wide range of renal insufficiency severity.

The association between the T280M and V249I polymorphisms of the CX3CR1 gene and renal insufficiency has already been hypothesized by two previous cross-sectional studies^25,26^. The first study was conducted in an Indian population and compared the prevalence of two polymorphisms in a group of 123 patients with advanced CKD (eGFR 13 ± 10 ml/min) and a group of 100 healthy controls^25^. The second study, conducted in an Egyptian population, examined T280M and V249I in a group of 100 patients with end-stage renal disease (ESRD) undergoing dialysis, compared to 100 healthy controls^26^. In both studies the M allele of the T280M genotype and the I allele of the V249I genotype were more frequent in patients with ESRD compared to healthy controls. Our results are consistent with findings related to the T280M polymorphism, whereas the association with the V249I is not confirmed.

In recent years, numerous studies have investigated the role of CX3CR1 polymorphisms in the progression of atherosclerosis^22,27–30^. The M allele of T280M has been associated with a lower prevalence of acute coronary events^27^, and a population-based study reported a reduction in cardiovascular risk^22^. The M allele is also linked to a slower progression of carotid atherosclerosis and plays a stabilizing role in plaque development at this site^29,30^, while it doesn’t show the same trend against peripheral arterial disease^31^. Surprisingly, a case-control study comparing CX3CR1 polymorphisms between 510 patients with cerebral infarction and 510 controls revealed an association between the M allele for T280M and an increased cerebrovascular risk^32^. This finding is consistent with the results from our study regarding the progression of CKD.

The apparent paradox in the effect of T280M-CX3CR1 on cardiovascular risk on the one hand, and cerebrovascular and renal damage risk on the other, has already been observed for other genes. In particular for T374A polymorphism of RAGE the presence of the A allele is associated with a reduced risk of ischemic heart disease^33^, while some cross-sectional studies showed an association of the A allele with CKD in diabetic subject^34,35^and carriers of this allele are shown to be at a higher risk of renal insufficiency progression^9^. Although the underlying mechanism is not yet clear, these seemingly contradictory findings regarding the T374A-RAGE polymorphism mirror what our study demonstrates about the T280M-CX3CR1 polymorphism.

Among the enrolled patients, those wild-type for T280M-CX3CR1 were more frequently treated with statins, fibrates, and urate-lowering drugs compared to carriers of the M allele (Table 2). The lipid profile values recorded at baseline showed no significant differences between the two groups (Table 1). In line with previously published data, this association suggests a potential anti-atherogenic role and cardiovascular risk reduction for the M allele of the T280M-CX3CR1 polymorphism. Nevertheless, multivariate analysis including the T280M polymorphism and treatment with lipid-lowering and urate-lowering drugs confirmed the significance of T280M-CX3CR1 in predicting the initiation of KRT. In this analysis, neither drug category reached statistical significance.

The fact that renal function values, as indicated by baseline creatinine and corresponding eGFR, are significantly different between the two groups is crucial for interpreting the results of this study. The homogeneity of the two groups in all other characteristics suggests that this difference is not due to a sampling bias but is more likely due just to the genetic profile of CX3CR1 polymorphisms carriers, which stratifies patients by renal insufficiency values. Indeed, genetic characteristics can be considered a priori compared to biochemical data; this becomes even more compelling when we correlate creatinine and eGFR values with the number of mutated alleles (Fig. 3). Although these differences across the three subgroups are appreciable, they only approach statistical significance. Notably, this finding is also confirmed by the multivariate analysis including eGFR at baseline and other clinical determinants of CKD progression, in which the association between T280M-CX3CR1 polymorphisms and outcome remains significant even after adjusting for renal function values at enrollment.

The relationship between T280M-CX3CR1 polymorphisms and renal function in our sample is further explored in Fig. 4. The progression of CKD secondary to nephroangiosclerosis or diabetes mellitus depends on various factors, with the expected reduction in eGFR typically estimated at 1.5–2 ml/min/m^2^ per year^36^. For wild-type patients in our sample, the distribution of calculated eGFR values at enrollment appears linear and aligns with this model, showing an estimated annual reduction of 1.52 ml/min/m^2^. In contrast, for patients with heterozygous or homozygous mutations, no relationship between age and eGFR at enrollment was observed. This suggests a less pronounced age effect, again indicating the impact of the T280M polymorphism on renal function.

This trend is more pronounced in the subgroup of younger patients. Table 3 presents patients aged ≤ 65 years at enrollment, categorized by eGFR stages. Patients in group 1 (*n* = 37) are distributed as expected, while patients in group 2 (*n* = 13) show a more homogeneous distribution. When comparing the two groups based on the number of patients who started dialysis during the observation period, a statistically significant difference emerges, to the disadvantage of group 2 (OR 5.48, 95% CI 1.29 to 23.1; *p* = 0.02). In younger patients, the presence of the T280M mutation is associated with an increased risk of progressing to dialysis, with a clear trend observed across all eGFR stages, even at the highest filtration values.

**Table 3:** *Comparison of eGFR classes in patients aged ≤ 65 years at enrollment*,* classified into wild-type (Group 1) and T/M + M/M mutation carriers (Group 2) for the CX3CR1 genotype. For each subgroup*,* the proportion of patients starting dialysis is also reported and compared.*

CKD stage	Group 1 (T/T)		Group 2 (T/M + M/M)		
	< 65 years	dialysis	< 65 years	dialysis	CHI-SQUARE
	*n* (%)	ratio (%)	*n* (%)	ratio (%)	OR (95% CI), *p*
Stage 1–2	23 (62%)	0/23 (0.00)	4 (31%)	1/4 (0.25)	20.1 (0.67 to 598.7), *p* 0.08
Stage 3a	9 (24%)	4/9 (0.44)	2 (15%)	1/2 (0.50)	1.25 (0.06 to 26.9), *p* 0.88
Stage 3b-5	5 (14%)	1/5 (0.20)	7 (54%)	4/7 (0.57)	5.33 (0.37 to 75.7), *p* 0.21
TOTAL	37 (100%)	5/37 (0.13)	13 (100%)	6/13 (0.46)	5.49 (1.29 to 23.2), *p* 0.02

It is important to recognise that the limited sample size substantially reduces the statistical power of these analyses, and as such, the observed trends should be interpreted with caution. Nevertheless, despite their exploratory and underpowered nature, these findings may offer insights that could support biological plausibility and help contextualize the longitudinal data related to the primary outcome.

The role of CX3CR1 gene polymorphisms in the progression of renal damage has been hypothesized in the literature, primarily linked to the recruitment and activation of fibroblasts and mononuclear monocytes within the renal parenchyma^37^. Exposure to Reactive Oxygen Species (ROS) increases CX3CR1 expression in renal fibroblasts, enhancing their migration in response to CX3CL1 and promoting their pro-fibrotic activity in the renal parenchyma^38^. Among renal cell types mononuclear phagocytes exhibit the highest levels of CX3CR1 expression, and this receptor has been shown to mediate their homing to the kidney^39–41^. In subjects with the M allele of the CX3CR1-T280M polymorphism, in vitro studies have shown reduced monocyte survival compared to wild type individuals^42^, along with increased cellular adhesion capacity^43^. This suggests a complex interaction between CX3CR1 genetics and inflammatory cells, where the same polymorphism can differentially affect the migration, adhesion, and survival of mononuclear phagocytes.

Recently, monocytes have been more precisely categorized into four subgroups based on receptor expression, with the intermediate monocytes subgroup characterized by high HLA-DR expression (HLA-DR^hi^) showing the highest levels of CX3CR1 expression^44^. Cormican and colleagues demonstrated an expansion of the HLA-DR^hi^ intermediate monocytes subgroup in patients with renal insufficiency, both in circulation and within the kidney. Additionally, their study showed that all monocyte subtypes from CKD patients present in vitro increased adherence to endothelial cells compared to those from healthy controls^45^.

Overall, these findings support the results of our study and provide a potential pathogenic hypothesis for the association between the T280M polymorphism and the progression of renal insufficiency.

As previously highlighted, the main strength of this study is its longitudinal design with extended follow-up. The enrolled patients exhibited a wide range of baseline renal function, and the cohort was homogeneous in terms of the etiology of renal insufficiency. Although renal biopsy was not performed, the exclusion criteria applied at enrollment allowed for the selection of patients likely to have CKD secondary to hypertensive and/or diabetic nephropathy. Finally, the chosen outcome, initiation of KRT, is a reliable indicator of renal insufficiency progression. Our data regarding the role of T280M-CX3CR1 align with previously published cross-sectional studies, confirming its potential as a target not only for diagnostic purposes but also for therapeutic investigation in future and larger studies.

The primary limitation of the study is the small sample size, which significantly reduces the statistical power to detect potential associations. Although the relationship between the T374A RAGE polymorphism and CKD progression was reported to be significant in a previous study on this same cohort^9^, the current analysis failed to confirm this association. This discrepancy likely reflects the combination of a limited number of events and a hard outcome definition, namely the initiation of KRT. Even the analysis using aggregated genotype data (wild-type vs. heterozygous and homozygous) resulted in a borderline p-value (p 0.066), suggesting that a larger cohort might have uncovered a significant effect. The issue is further compounded in the analysis of the V249I-CX3CR1 polymorphism where data were unavailable for 23 of the 121 patients, further reducing the sample size therefore contributing to the overall low power of the analyses involving this polymorphism. Additionally, the allele distribution in our population (Fig. 1) diverges from previous reports, possibly due to ethnic differences (Caucasian vs. Indian and Egyptian cohorts), but the limited number of observations prevents robust subgroup analyses.

Another limitation of our study is the absence of intermediate renal function evaluations throughout the follow-up. In our setting, longitudinal creatinine data were primarily collected for clinical indications, and thus only available in patients with more severe disease trajectories. This inherent selection bias prevents the use of surrogate markers such as the decline or halving of eGFR, and restricts the ability to model disease progression over time. Moreover, pharmacological treatment was available only at baseline, and the lack of longitudinal data on therapy and adherence precluded adjustment for treatment effects on renal outcomes. Together, these limitations reduce the generalizability of our findings.

The results of this study corroborate the role of the CX3CR1 T280M polymorphism in the progression of renal insufficiency. Specifically, carriers of the M allele have an increased risk of developing ESRD requiring KRT. This finding is consistent with previous observational studies on CX3CR1 polymorphisms and preclinical studies on the role of monocytes in the pathogenesis of renal insufficiency. However, our study does not confirm the association between the V249I-CX3CR1 or T374A-RAGE polymorphisms and the progression of CKD. Genetic analysis of CX3CR1 may be crucial for determining the prognosis of patients with renal disease from its earliest stages, and represents a promising area for future research aimed at identifying potential therapeutic targets.

## Materials and methods

This was a prospective observational study. We included patients affected by mild to severe CKD from the PLIC study (“Progressione delle Lesioni Intimali Carotidee”), a population-based cohort representative of the general population of the northern area of Milan^46^. All individuals were enrolled through the outpatient clinic of the Nephrology and Dialysis Unit of Bassini Hospital (Milan, Italy) from 1 st January 2005 to 1 st February 2005.

The exclusion criteria for the enrollment were: inflammatory or infectious disorders, congenital or hereditary kidney diseases, previous dialysis, glomerulonephritis and cardiovascular events (i.e. acute myocardial infarction, acute heart failure, stroke, transient cerebral ischaemic attack, acute coronary syndrome and chronic heart failure New York Heart Association NYHA class III and IV) in the previous 6 months, and microvascular complications (including diabetic foot ulcer), previous major surgery (i.e. carotid thrombo-arterectomy, percutaneous coronary angioplasty, arterial angioplasty or arterial bypass of the lower limbs, coronary bypass and amputations), malignant neoplasms and thyroid dysfunction.

For all participants, medical histories were obtained, blood pressure was measured and BMI was calculated (in kg per m^2^). Blood and urine samples, both early morning and 24-hours, were collected. Estimated glomerular filtration rate (eGFR) was established using the Chronic Kidney Disease Epidemiology Collaboration 2021 (CKD-EPI 2021) equation based on baseline creatinine levels. CKD was properly classified using current guidelines^47^. Genomic DNA was extracted from cells in the buffy coat isolated from samples collected after overnight fasting using the Flexigene DNA kit (Qiagen, Milan, Italy) according to the manufacturer’s protocol^9^. Genotyping for the − 374 T/A RAGE polymorphism was performed on 1 mL (10 to 200 ng of DNA), using a TaqMan allelic discrimination test. Genotyping for the V249I (rs3732379) and T280M (rs3732378) polymorphisms was performed as described^27^. Real-time PCR was carried out using a standard SYBR Green PCR kit (Qiagen, Hilden, Germany).

During the observation period, initiation events of kidney replacement therapy (KRT) were recorded as primary outcome. These events were defined as the commencement of chronic dialysis therapy, either hemodialysis or peritoneal dialysis, or the performance of renal transplantation. The decision to initiate dialysis was made on a clinical basis in patients whose eGFR had declined below 15 ml/min/m^2^. Importantly, patients who underwent conservative management of ESRD without initiation of dialysis or transplantation were not counted toward the primary outcome.

No patients were lost to follow-up. Among the thirty-five patients who discontinued follow-up at our center during the study period, the primary outcome was ascertained through regional electronic health records. One patient initiated KRT at an external center and was counted as an event; the remaining patients without evidence of KRT initiation were censored at the time of their last available clinical assessment.

The PLIC Study has been approved by the Ethics Committee of the University of Milan (SEFAP/Pr.0003). Written informed consent was obtained from all the subjects included in the study (all over 18 years-old), for the use of their biological samples and health data subsequently to their collection during the first evaluation of the study, their anonymized information to be published in this article. All methods were in accordance with the Declaration of Helsinki.

### Statistical analysis

The continuous variables were expressed as Mean ± Standard Deviation [Confidence Interval]. The patient cohort was stratified based on the T280M polymorphism into two groups: Group 1 consisting of wild-type T/T patients (87 patients, 71.9%), and Group 2 comprising heterozygous T/M (31 patients, 25.6%) and homozygous M/M patients (3 patients, 2.5%). Due to the limited sample size, particularly the very low number of homozygous individuals (3 patients), heterozygous and homozygous patients were combined into a single group (Group 2) for statistical analyses.

Independent samples T-test was used to test for baseline differences between the two groups for normally distributed variables, and Mann-Whitney U-test for non-normally distributed variables. The distribution of sexes, smoke habitude, diabetes, history of cardiovascular events and medications among the two groups was assessed with a chi-square. The significance was assumed for p-values < 0.05.

Survival analysis was first explored using Kaplan-Meier curves, with differences between groups assessed by the log-rank test. Subsequently, survival analysis was performed using Cox proportional hazards multivariate analysis, with the outcome defined as the initiation of KRT. The following models were generated: univariates and multivariate analyzing genetic polymorphism of CX3CR1-T280M, V249I and RAGE-T374A. The significance was assumed for p-values < 0.05.

A cross-sectional analysis was performed to investigate the association between age and eGFR at baseline. A linear regression model was applied with eGFR as the dependent variable and age, group, and their interaction term (age × group) as independent variables. The model estimates were reported with 95% Confidence Intervals and p-values.

Statistical analysis was performed using SPSS software, Version 22 (IBM, Inc., Chicago, IL, USA).

## Data Availability

The datasets generated and/or analysed during the current study are not publicly available due to their sensitive nature and the potential risk of participant re-identification. However, de-identified individual participant data are available from the corresponding author (Matteo Gatti, MD; dott.matteogatti@gmail.com) upon reasonable request. Data access will be granted for research purposes only, following evaluation of the request and in compliance with applicable ethical and data protection regulations.
